# BH3-mimetics and BET-inhibitors elicit enhanced lethality in malignant glioma

**DOI:** 10.18632/oncotarget.16365

**Published:** 2017-03-18

**Authors:** Chiaki Tsuge Ishida, Elena Bianchetti, Chang Shu, Marc-Eric Halatsch, M. Andrew Westhoff, Georg Karpel-Massler, Markus D. Siegelin

**Affiliations:** ^1^ Department of Pathology & Cell Biology, Columbia University Medical Center, New York, New York, USA; ^2^ Department of Neurosurgery, Ulm University Medical Center, Ulm, Germany; ^3^ Department of Pediatrics and Adolescent Medicine, Ulm University Medical Center, Ulm, Germany

**Keywords:** apoptosis, BH3-mimetics, brain cancer, c-myc, Bcl-xL

## Abstract

Drug combination therapies remain pivotal for the treatment of heterogeneous malignancies, such as glioblastomas. Here, we show a novel lethal interaction between Bcl-xL and c-myc inhibition accomplished by bromodomain protein inhibitors. Established, patient-derived xenograft and stem cell-like glioma cells were treated with the novel bromodomain protein inhibitors, JQ1 and OTX015, along with BH3-mimetics, ABT263 or Obatoclax. Synergy was assessed by calculation of CI values. Small interfering RNAs (siRNAs) were used for gene silencing and mechanistic studies. *In vivo* experiments were performed in a glioblastoma xenograft model. Single treatments with JQ1 and OTX015 had only moderate effects on the reduction of cellular viability. However, the combination treatment of BH3-mimetics along with JQ1 or OTX015 resulted in a highly synergistic reduction of cellular viability in a broad range of different model systems of malignant glioma. Similarly, knockdown of c-myc sensitized glioma cells for ABT263 mediated cell death. The enhanced loss of cellular viability in the combination treatment was mediated by activation of apoptosis with dissipation of mitochondrial membrane potential and caspase cleavage. The combination treatment led to a modulation of anti- and pro-apoptotic Bcl-2 family members with an increase in pro-apoptotic Noxa mediated by ATF4. Small interfering RNA mediated knockdown of Bak and Noxa protected glioma cells from ABT263/JQ1 mediated apoptosis. Finally, the combination treatment of ABT263 and OTX015 resulted in a regression of tumors and a significantly smaller tumor size as compared to single or vehicle treated tumors. Thus, these results warrant clinical testing for the drug combination of BH3-mimetics along with bromodain protein inhibitors.

## INTRODUCTION

Glioblastoma and malignant gliomas remain incurable diseases [1]. It is for that reason that new therapeutic approaches need to be elucidated. It is well established that malignant gliomas are one of the most heterogeneous tumor entities. Therefore, many oncogenic pathways are simultaneously active in one tumor [2]. The activation of these pro-survival pathways results in dysregulation of apoptosis with high levels of inhibitor of apoptosis proteins, such as XIAP, cIAP-1 and survivin, and anti-apoptotic Bcl-2 family members, which in turn block physiological cell death and consequently promote unrestrained growth. Most notably, Bcl-2 family members, such as Bcl-2, Bcl-xL and Mcl-1, are expressed at high levels in gliomas, rendering these molecules potential targets for therapy. This has led to the discovery and synthesis of a certain class of molecules, which are known as BH3-mimetics due to their high affinity binding to anti-apoptotic Bcl-2 family members.

Amongst the oncogenic transcription factors c-myc is known to regulate cell death and was considered for a long-time to be “undruggable” [3]. However, Bromodomain and Extra-Terminal proteins (BET), such as BRD2 and BRD4 were shown to modulate c-myc mRNA and protein levels and therefore were identified as potential targets to interfere with c-myc [4]. Very recently, BET inhibitors, such as the thienotriazolodiazepines, JQ1 and the derivative OTX015, were shown to suppress c-myc expression at the level of transcription [5]. Consequently, c-myc-dependent tumors, such as Burkitt-lymphoma and its associated preclinical model systems display marked sensitivity against BET inhibitors [6]. Aside from hematological malignancies, solid tumors, such as malignant gliomas, are sensitive to BET inhibitors as well [5]. However, the anti-glioma activity of BET inhibitors appears to be limited [5]. That's why new combination therapies need to be identified to enhance the efficacy of BET inhibitors.

In this work, we identified a synthetic lethal interaction between Bcl-xL and c-myc inhibition in various model systems of malignant glioma, including patient-derived xenograft and stem cell like glioma cells. Notably, stem cell-like glioma cells display a remarkable sensitivity to the drug combination of BET-inhibitors, such as JQ1 and OTX015, and BH3-mimetics.

## RESULTS

### Treatment with JQ1 elicits antiproliferative activity in glioblastoma cells

**Figure 1:**
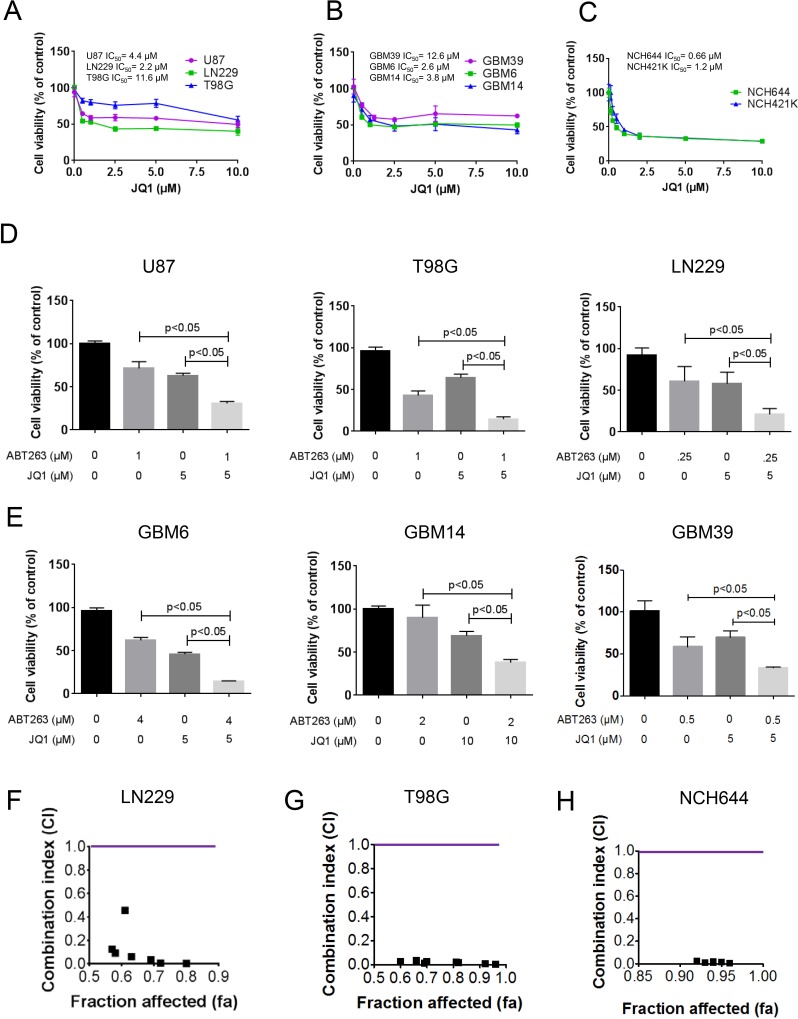
Combined treatment with ABT263 and the BET-inhibitor (JQ1) results in a synergistic antiproliferative effect across a wide spectrum of human glioma cells **A**.-**C**. U87MG, LN229, T98G established glioblastoma cell lines, GBM39, GBM6 and GBM14 patient-derived xenograft cultures and NCH644 and NCH421k stem cell-like glioma cultures were treated as indicated with JQ1. After 72h of treatment, CellTiter-Glo assays were performed. IC_50_ values were calculated. Column: mean. Error bar: standard deviation (SD). *n* = 3. **D**., **E**., U87MG, LN229, T98G established glioblastoma cell lines, GBM39, GBM6 and GBM14 patient-derived xenograft cultures were treated with ABT263, JQ1 or the combination of both. After 72h of treatment, CellTiter-Glo assays were performed. Column: mean. Error bar: standard deviation (SD). *n* = 3. Statistical analysis was performed and *p* values were calculated. A p-value of less than 0.05 was considered statistically significant. **F**.-**H**., LN229, T98G and NCH644 glioblastoma cells were treated for 72 hours with ABT263, JQ1 or the combination and analyzed by CellTiter-Glo assay. CI values and fraction affected were calculated using the CompuSyn software (ComboSyn, Inc., Paramus, NJ, U.S.A.). Data points located below 1 (CI value less than 1) indicate a synergistic drug-drug interaction and data points larger than 1 indicate an antagonistic drug-drug interaction. Some data points overlap and are therefore not represented on the graphical chart. A colored line highlights CI value 1. For individual values, please refer to Table 1.

In order to assess the effects of JQ1 on the proliferation of glioblastoma cells, we treated established glioblastoma cells of different genetic backgrounds (U87, LN229 and T98G), patient derived xenograft cells (GBM6, GBM14 and GBM39) and stem cell-like glioma cells (NCH644 and NCH421k) with increasing concentrations of JQ1 (Figure 1A-1C). After 72h, cells were subjected to proliferation assays (CellTiter-Glo®-assays). JQ1 displayed a dose-dependent response in all cell lines tested (Figure 1A-1C). Notably, stem cell-like glioma cells appeared to be more sensitive than established and patient-derived xenograft cells. However, high concentrations of 10 μM of JQ1 did not result in complete growth inhibition, suggesting that the effects of JQ1 might be enhanced through the addition of another compound.

### The combination treatment of ABT263 and JQ1 elicits synergistic anti-proliferative effects

Based on the fact that c-myc inhibition has an impact on intrinsic apoptosis, we hypothesized that JQ1 and ABT263 [7] might synergistically act on tumor cell growth. To test this hypothesis, established glioblastoma cells (U87, T98G and LN229) cells were treated with JQ1, ABT263 or the combination of both compounds. After 72h, viability assays were performed. We found that the combination treatment resulted in a potent reduction of cellular viability in a statistically significant manner (Figure 1D). Similar results were obtained in patient-derived xenograft lines (GBM6, GBM14 and GBM39) (Figure 1E) and in stem-cell like glioma cells (NCH644 and NCH421k) (Figure 1H and Supplementary Figure 1B). To prove that the combination treatment reduces cellular viability of glioma cells in a synergistic manner, we calculated combination index (CI) values for the drug combination of ABT263 and JQ1 in LN229, T98G, NCH421k and NCH644 cells. All concentrations tested resulted in highly synergistic CI values (significantly below 1) (Figure 1F-1H, Supplementary Figure 1B and Table 1). We verified as to whether or not structural similar compounds, such as OTX015, were capable of enhancing reduction of cellular viability mediated by ABT263. Akin to the effects of JQ1, the drug combination of OTX015 and ABT263 was significantly more effective than OTX015 or ABT263 alone (Supplementary Figure 1A).

**Table 1:** CI values for glioblastoma cultures after combinatorial treatments with ABT263 and JQ1

T98G			LN229			NCH644		
ABT263 (μM)	JQ1 (μM)	CI	ABT263 (μM)	JQ1 (μM)	CI	ABT263 (μM)	JQ1 (μM)	CI
0.25	5	0.01428	1.0	5	0.00373	1.0	10.0	0.02111
0.5	4.0	0.02653	0.5	2.5	0.00186	2.0	8.0	0.01897
1.0	3.0	0.02098	0.25	1.0	0.00679	3.0	5.0	0.00564
2.0	2.0	0.00294	0.125	0.5	0.09112	4.0	3.0	0.02502
2.0	5.0	0.00911	0.125	5.0	0.45551	4.0	10.0	0.01669
1.0	4.0	0.01894	0.25	2.5	0.03430	3.0	8.0	0.00758
0.5	3.0	0.03524	0.5	1.0	0.06114	2.0	5.0	0.01316
0.25	2.0	0.02639	1.0	0.5	0.12614	1.0	3.0	0.01122

ABT263 treatment elicits a synergistic anti-proliferative effect on LN229, T98G glioma cells and NCH644 stem cell-like glioma cells in the presence of the c-myc inhibitor, JQ1. The CompuSyn software (ComboSyn, Inc., Paramus, NJ) was used for the drug-drug interaction analysis including the calculation of the combination index (CI). A CI<1 was considered as synergistic, a CI=1 as additive and a CI>1 as antagonistic.

### The combination treatment of ABT263 and JQ1/OTX015 resulted in enhanced apoptotic cell death

**Figure 2:**
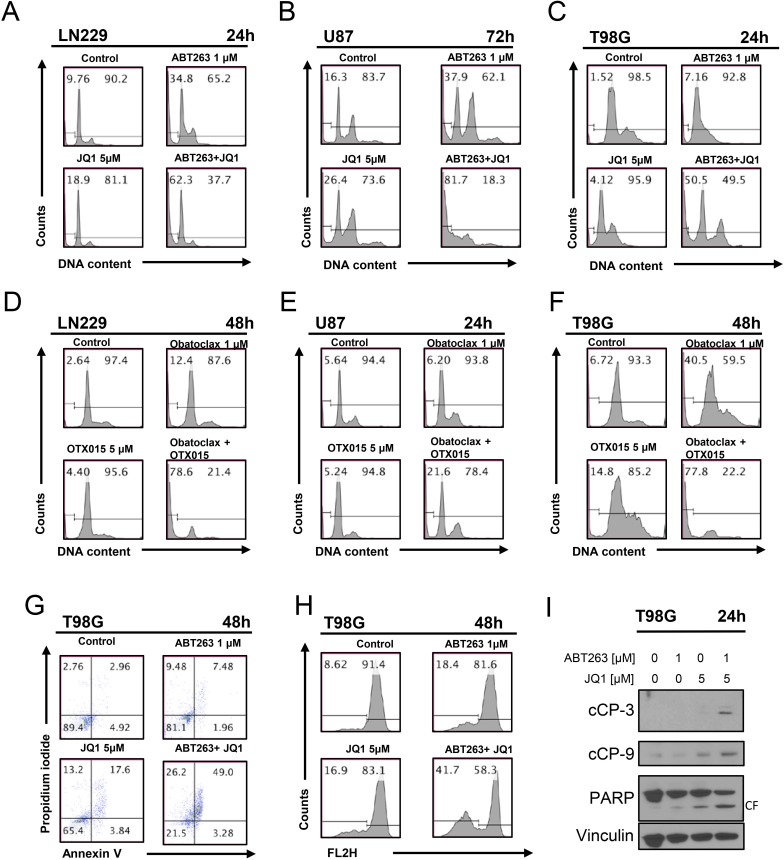
Combined treatment with JQ1 and ABT263 yields enhanced induction of apoptosis **A**., **B**., **C**. representative histograms of LN229, U87 and T98G glioblastoma cells that were treated for the indicated time points as indicated with JQ1, ABT263 or both prior to staining with propidium iodide and flow cytometric analysis. **D**.-**F**., LN229, U87 and T98G glioblastoma cells were treated for the indicated time points as indicated with OTX015, Obatoclax or the combination. **G**., representative histograms of T98G glioblastoma cells treated with ABT263, JQ1 or the combination as indicated for 48h prior to staining for annexin V and propidium iodide and flow cytometric analysis. **H**., representative histograms of T98G glioblastoma cells that were treated with JQ1, ABT263, or both prior to staining with TMRE and flow cytometric analysis. **I**., T98G glioblastoma cells were treated for 24 h with JQ1, ABT263 or the combination. Whole-cell extracts were examined by Western blot analysis for PARP, cleaved caspase 9 (C9) and cleaved caspase 3. Vinculin Western blot analysis was performed to confirm equal protein loading.

Given the morphological appearance of the cells with blebbing and fragmentation, we hypothesized that the combination treatment of ABT263 and JQ1 kills cells by enhanced apoptosis. To this end, we treated LN229, T98G and U87 cells with ABT263, JQ1 or the combination and analyzed them for DNA – fragmentation by flow cytometry. In keeping with the results from the viability assays, the combination treatment led to a significant higher amount of DNA – fragmentation as compared to single treatments and control (Figure 2A-2C). In order to confirm that not only ABT263 synergizes with c-myc inhibitors, we assessed also the effects on apoptosis with another BH3-mimetic, Obatoclax. In agreement with the results above, the combination treatment of Obatoclax and OTX015 led to a significant enhancement of apoptosis as compared to each drug alone (Figure 2D-2F). To further support an apoptotic cell death, T98G and GBM14 cells were treated in a similar fashion as above and subsequently stained with annexin V and propidium iodide. Similar to the DNA – fragmentation results, the combination treatment led to more annexin V positive cells as compared to single treatments (Figure 2G and Supplementary Figure 2A).

### ABT263 and JQ1 as well as ABT263 and OTX015 led to enhanced dissipation of mitochondrial membrane potential in glioma cells

Based on the hypothesis that ABT263 and JQ1 enhanced intrinsic apoptosis, we determined as to whether or not the combination treatment of ABT263 and JQ1 as well as ABT263 and OTX015 cause enhanced dissipation of mitochondrial membrane potential. To this purpose, T98G cells were treated with ABT263, JQ1 or OTX015 or the combination and subsequently stained with TMRE to assess mitochondrial membrane potential. In agreement with earlier results on apoptosis induction, the combination treatment of ABT263 and JQ1 as well as ABT263 and OTX015 led to a more pronounced dissipation of mitochondrial membrane potential than each compound on its own (Figure 2H and Supplementary Figure 2B), suggesting that intrinsic apoptosis might be involved.

### The combination treatment of ABT263 and JQ1 elicits activation of effector- and initiator caspases

To determine if caspases are involved in the combination treatment of ABT263 and JQ1, we assessed cleavage of caspase-3, caspase-9 and PARP. T98G cells treated with ABT263 and JQ1 displayed stronger activation of initiator-caspase-9 and effector-caspase-3 (Figure 2I). In agreement with these findings, cleavage of the DNA-repair enzyme PARP, which is specific for apoptosis detection (89 kDa product) was enhanced in the combination treatment, suggesting that activation of caspases is part of the cell death mechanism (Figure 2I). Similar results were seen in LN229 glioblastoma cells (Supplementary Figure 2C). To demonstrate that caspases are involved in ABT263/JQ1 mediated apoptosis, we treated LN229 with ABT263+JQ1 in the presence or absence of the pancaspase inhibitor, zVAD-fmk. Our results show that zVAD-fmk protects cells from DNA – fragmentation induced by ABT263+JQ1 (Supplementary Figure 2D), suggesting that apoptosis and caspases play a role in the death mediated by the combination treatment.

### The c-myc inhibitor, JQ1, regulates the expression of Bcl-2 family members with down-regulation of Mcl-1 and up regulation of Noxa

**Figure 3:**
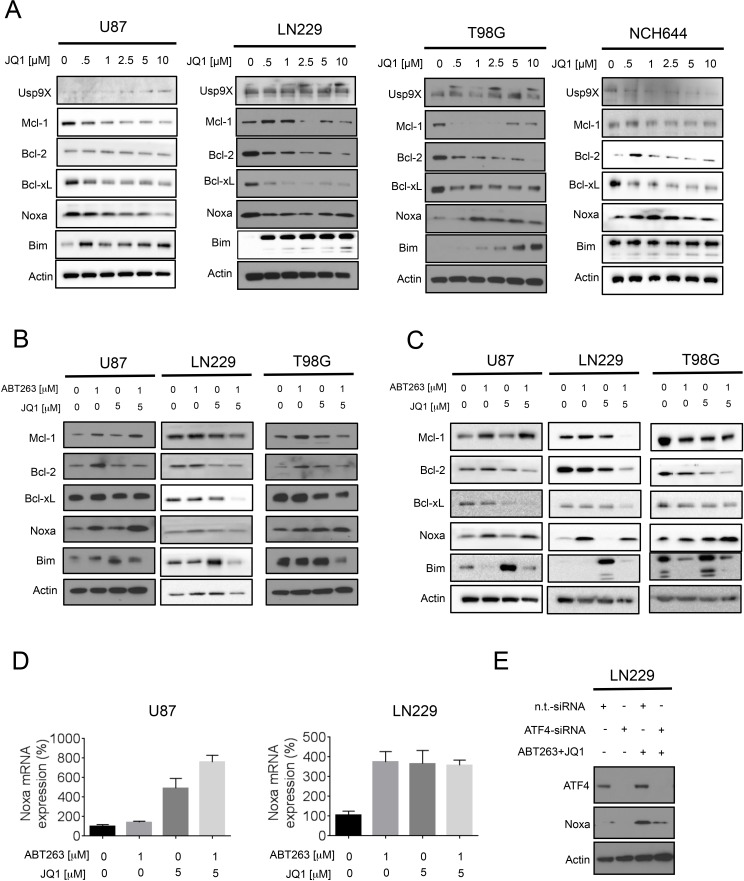
Treatment with JQ1 and the combination treatment (ABT263+JQ1) modulates protein expression of the Bcl-2 family of proteins **A**., Established glioblastoma cells, U87,LN229, T98G and stem cell-like glioma cells, NCH644 were treated with increasing concentrations of JQ1 as indicated for 72h. Whole cell extracts were collected and Western blot analysis was performed for Usp9X, Mcl-1, Bcl-2, Bcl-xL, Noxa and Bim. Actin served as loading control. **B**., **C**. U87, LN229 and T98G cells were treated with ABT263, JQ1 or the combination of both for 24h (B) and 48h (C). Whole cell extracts were collected and Western blot analysis was performed for Mcl-1, Bcl-2, Bcl-xL, Noxa and Bim. Actin served as loading control. **D**., U87 and LN229 cells were treated with ABT263, JQ1 or the combination of both. After 7h cells were harvested and RNA was isolated. Real-time PCR for Noxa was conducted and the results were normalized to 18S. Column: mean. Error bar: standard error of measurement (SEM). G, LN229 cells were transfected with non-targeting (n.t.) siRNA or an ATF4 specific siRNA. 72h after transfection cells were treated with ABT263 and JQ1 for 7h. Cells were harvested and analyzed by western blotting for ATF4 and Noxa. Actin serves as loading control.

In order to provide an explanation as to why the combination treatment of ABT263 and JQ1 enhances apoptosis and caspase-cleavage, we hypothesized that JQ1 might affect the levels of the anti- and pro-apoptotic Bcl-2 family members in malignant glioma cells. To this purpose, LN229, T98G, U87, NCH644 and GBM6 glioblastoma cells underwent treatment with increasing concentrations of JQ1 for 72h (Figure 3A and Supplementary Figure 3). Subsequently, the lysates were analyzed for protein expression by western blotting. In all cell lines tested, we found a dose dependent decrease of Bcl-xL and Mcl-1. Bcl-2 was down in LN229, T98G and GBM6, but no modulation was seen in U87 and NCH644 cells. The deubiquitinase Usp9X, which interacts with and stabilizes Mcl-1, was not significantly altered by JQ1 in T98G, LN229, U87 and GBM6 cells, suggesting that JQ1 mediated down-regulation of Mcl-1 is independent of Usp9X. However, JQ1 suppressed Usp9X protein levels in NCH644 cells. With regards to the pro-apoptotic Bcl-2 family members, we noted a consistent increase of BIM levels in all cell cultures tested. In contrast, Noxa was modulated by JQ1 in a less consistent fashion with an increase in T98G and NCH644 cells, a transient decrease in LN229 and a dose-dependent reduction in U87 cells. We were unable to detect baseline expression of Noxa in GBM6 cells (Supplementary Figure 3), suggesting that these cells might express relatively low levels of Noxa under non-stressed conditions.

### The combination treatment of ABT263 and JQ1 elicits an increase of Noxa protein levels

Next, we assessed the impact of the combination treatment on the expression levels of the various Bcl-2 family members. While U87 cells show a mild increase in Mcl-1 levels after treatment with ABT263 and JQ1, LN229 and T98G cells revealed a suppression of Mcl-1 protein levels (at 24 and 48 h after treatment) (Figure 3B-3C). The combination treatment led to a suppression of Bcl-2 in U87, T98G and LN229 after 48h of treatment (Figure 3C). Bcl-xL and Bcl-2 were down in all cell cultures tested (T98G, LN229, U87) at 48h (Figure 3C). While BIM was up regulated only by single treatment with JQ1, the combination treatment increased the levels of Noxa in U87, LN229 and T98G cells at 48 h (Figure 3C), suggesting that Noxa might be a key player in the death induced by ABT263+JQ1. The increase in Noxa was earlier detected in T98G and U87 (already at 24 hours), but was also finally appreciated in LN229 cells (48h).

### The increase of Noxa protein mediated by ABT263 and JQ1 occurs at the levels of transcription and is mediated by the Activating Transcription Factor 4 (ATF4)

Noxa is known to be up-regulated in response to death stimuli at the level of transcription. To test the hypothesis that ABT263 and JQ1 mediate an increase in Noxa mRNA levels, we treated U87 and LN229 cells with ABT263, JQ1 or the combination of both for 7 hours and analyzed Noxa mRNA levels. We found that mRNA levels were increased in U87 and LN229 upon treatment with ABT263 and JQ1 (Figure 3D). While in LN229 cells ABT263, JQ1 and the combination of both elicited an almost equal increase of Noxa levels, the combination treatment showed the strongest increase in Noxa mRNA levels in U87 cells (Figure 3D). Given that we detected an increase in Noxa mRNA upon treatment with ABT263 and JQ1, we tested the hypothesis that ATF4 is implicated in ABT263-JQ1 mediated up regulation of Noxa levels. To this purpose, LN229 cells (mutated TP53) were transfected with a non-targeting siRNA or an ATF4 specific siRNA. After transfection, LN229 cells were treated with the combination treatment of ABT263 and JQ1 for 7h. Knockdown efficiency of ATF4 was confirmed in LN229 cells (Figure 3E). Compared to non-targeting siRNA transfected LN229 cells, ATF4-siRNA transfected LN229 cells displayed a significant attenuation of Noxa protein up-regulation upon administration of the combination treatment (Figure 3E), suggesting that ATF4 is a major driver of Noxa protein levels.

### Specific knockdown of Bcl-xL enhances JQ1 mediated apoptosis and recapitulates the effects of ABT263

**Figure 4:**
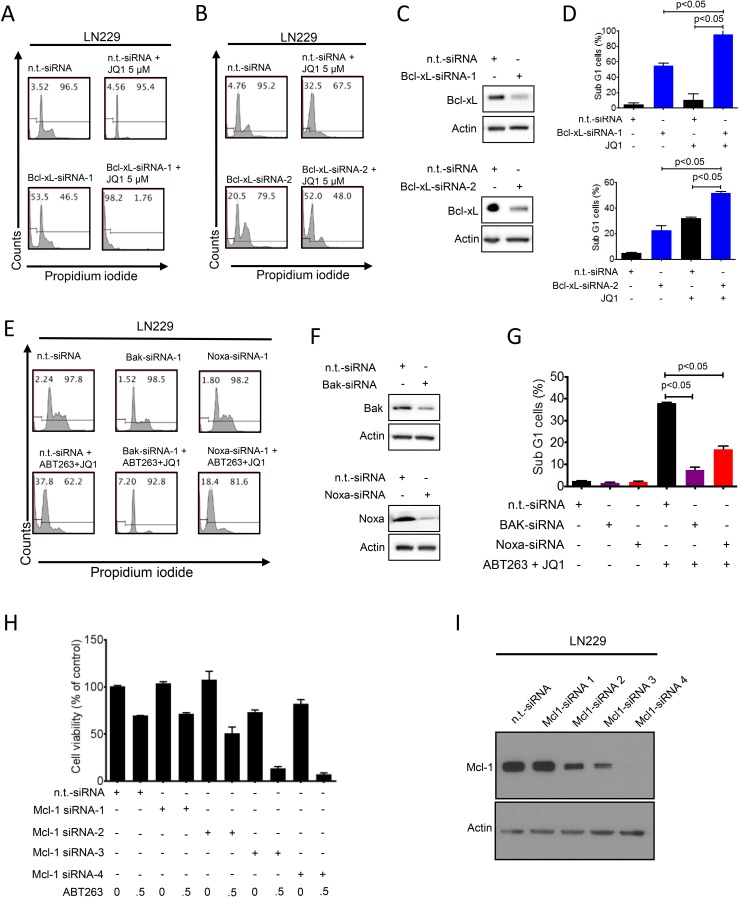
Functional implications of Bcl-2 family members in the combined treatment of ABT263 and JQ1 **A**.-**D**., Representative flow plots of LN229 cells that were treated with n.t.-siRNA or 2 different Bcl-xL siRNAs prior to additional treatment with either solvent or JQ1. Staining for propidium iodide and flowcytometric analysis was performed to determine the fraction of subG1 cells. The results were quantified (D). Knockdown of Bcl-xL was confirmed by Western Blot analysis (C). Actin served as loading control (C). **E**.-**G**. LN229 cells were treated with n.t.-siRNA or Noxa-siRNA or Bak-siRNA prior to treatment with solvent or the combination of 1μM ABT263 and 5μM JQ1 as indicated for 24 h. Staining for propidium iodide and flow cytometric analysis was performed to determine the fraction of subG1 cells. Representative flow plots are shown (E). The quantifications are shown in (G). Noxa and Bak knockdowns were confirmed by Western blot analysis (F). **H**., LN229 cells were transfected with n.t.-siRNA or four individual Mcl-1 siRNAs for 48h. Subsequently, cells were treated with ABT263 and analyzed by CellTiter-Glo assay. The concentrations for ABT263 are in μM. Column: mean. Error bar: standard deviation (SD). **I**., LN229 cells were transfected as in G and analyzed for protein expression of Mcl-1. Actin served as a loading control.

Next, we confirmed specifically that Bcl-xL inhibition is the main target of ABT263 in the drug combination of BH3-mimetics and JQ1. To this purpose, we utilized two Bcl-xL specific siRNAs and transfected them into LN229 cells (Figure 4A-4D). Specific knockdown of Bcl-xL was confirmed by immunoblotting (Figure 4C). 48 h after transfection, LN229 cells were exposed to JQ1 and subsequently analyzed for DNA – fragmentation by flow cytometry. The effect on apoptosis by both Bcl-xL siRNAs was enhanced in the presence of JQ1 (*p* < 0.05) (Figure 4D), recapitulating the effects of the drug combination (Figure 4A-4B). These findings suggest that Bcl-xL is a pivotal factor in the drug combination of ABT263 and JQ1 and that ABT263 most likely contributes to the apoptotic effects of the drug combination by interfering with Bcl-xL.

### Knockdown of Bak and Noxa protects from apoptosis induced by the combination treatment of ABT263 and JQ1

Since we detected an increase in Noxa levels by the drug combination, we determined as to whether or not Noxa is a key factor in ABT263/JQ1 mediated apoptosis. To this end, we silenced the expression of Noxa by siRNA in LN229, which was confirmed by immunoblotting (Figure 4F). Suppression of Noxa protected from apoptosis induced by the drug combination (*p* < 0.05) (Figure 4ED and 4G). Given that Noxa acts on Mcl-1 and Mcl-1 avidly binds Bak, but not Bax, it was pivotal to assess the role of Bak in ABT263/JQ1 mediated cell death. To this purpose, we silenced the expression of Bak in LN229 and knockdown was confirmed by immunoblotting (Figure 4F). LN229 cells silenced for Bak showed significantly less induction of apoptosis induced by ABT263 and JQ1 as compared to cells that were transfected with non-targeting siRNA (*p* < 0.05) (Figure 4E and 4G).

### Knockdown of Mcl-1 is sufficient to enhance reduction in cellular viability mediated by ABT263

Given that Noxa inhibits the anti-apoptotic activity of Mcl-1 and the combination treatment of ABT263 and JQ1 led to a suppression of Mcl-1 protein levels, we assessed the importance of Mcl-1 in ABT263/JQ1 mediated cell death. In this context, LN229 cells were transfected with non-targeting or four siRNAs that target Mcl-1 and subsequently treated with ABT263. In the presence of ABT263, silencing of Mcl-1 led to an increased reduction of cellular viability as compared to non-targeting siRNA (Figure 4H), confirming that Mcl-1 is an important mediator of resistance towards ABT263. Notably, the amount of Mcl-1 reduction appeared to correlate with the sensitivity to ABT263 (Figure 4H-4I).

### Knockdown of c-myc mimics the effect of JQ1 or OTX015 and enhances ABT263 mediated apoptosis

**Figure 5:**
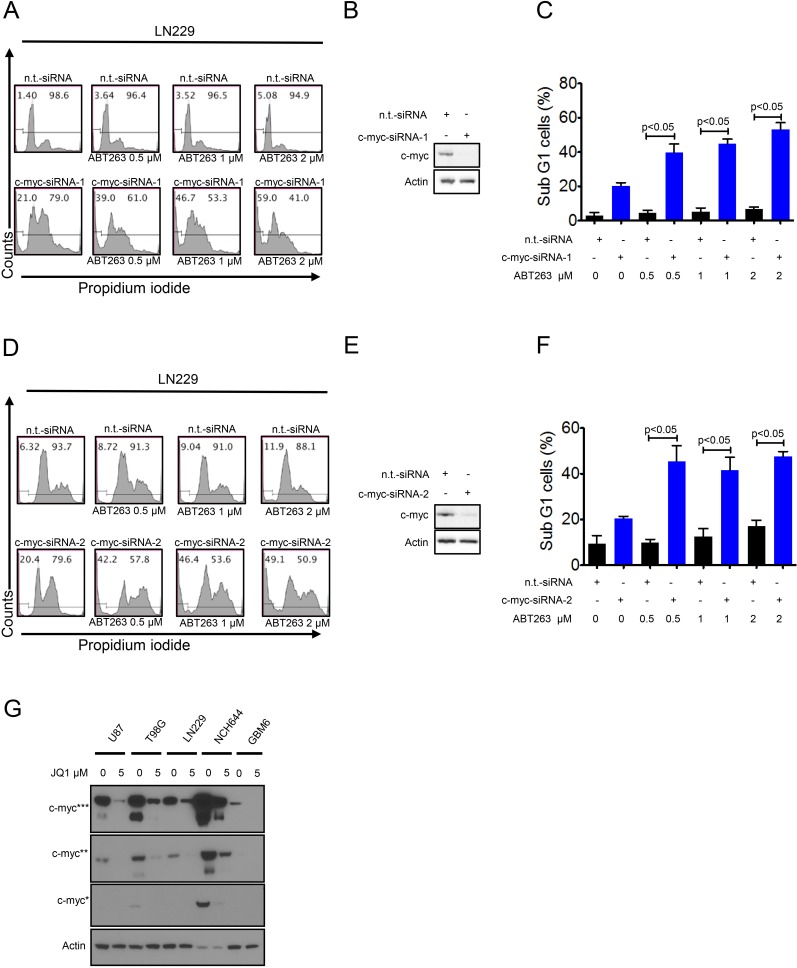
Silencing of c-myc sensitizes for Bcl-xL inhibition **A**., LN229 cells were transfected with n.t.-siRNA or c-myc-siRNA-1 prior to treatment with solvent or ABT263 as indicated for 24 h. Staining for propidium iodide and flow cytometric analysis were performed to determine the fraction of subG1 cells. Representative flow plots are shown. The quantifications are shown in **C**. c-myc knockdown was confirmed by Western blot analysis **B**. **D**., LN229 cells were transfected with n.t.-siRNA or c-myc-siRNA-2 prior to treatment with solvent or ABT263 as indicated for 24 h. Staining for propidium iodide and flow cytometric analysis were performed to determine the fraction of subG1 cells. Representative flow plots are shown. The quantifications are shown in **F**. c-myc knockdown was confirmed by Western blot analysis **E**. **G**. U87, T98G, LN229 (established glioblastoma cells), NCH644 (stem cell-like glioma cells) and GBM6 (patient-derived xenograft) cells were treated with JQ1 for 72 hours. Protein extracts were prepared and samples were analyzed by conventional western blot analysis for the expression of c-myc. Actin serves as a loading control. Multiple exposures are presented due to the fact that cells express different baseline levels of c-myc. * indicate different exposure times for c-myc protein.

Given that JQ1 and OTX015 are major inhibitors of c-myc [5], we hypothesized that these compounds enhance ABT263 mediated cell death mainly by suppression of c-myc protein levels. To verify this hypothesis, we specifically knocked down c-myc protein by two different siRNAs (Figure 5B and 5E). Knockdown of c-myc was confirmed by immunoblotting (Figure 5B and 5E). Silencing of c-myc (siRNA-1 and -2) enhanced ABT263 mediated cell death as compared to non-targeting siRNA (Figure 5A, 5C, 5D and 5F). These results and the previous observation above that silencing of Bcl-xL synergizes with JQ1 suggest that there is a synthetic lethal interaction between c-myc and Bcl-xL inhibition. Finally, we confirmed that JQ1 inhibits c-myc protein expression in established glioblastoma cell lines (U87, T98G, and LN229) as well as in stem cell-like (NCH644) and patient derived xenograft cultures (GBM6) (Figure 5G). Notably, c-myc protein levels are particularly high in stem cell-like glioma cells (Figure 5G), which is in keeping with earlier studies.

### The combination treatment of ABT263 and OTX015 results in growth reduction in a heterotopic model of glioblastoma

**Figure 6:**
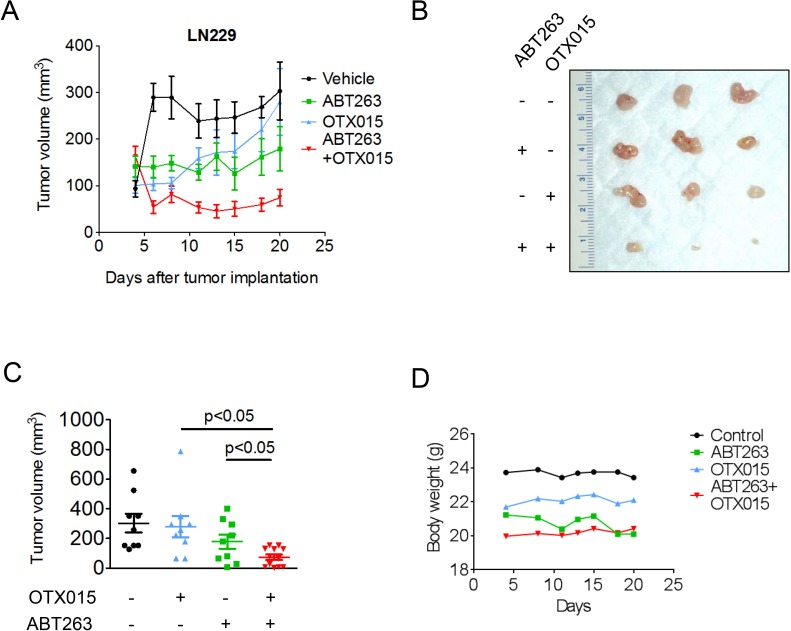
Combined treatment with ABT263 and OTX015 leads to a regression of glioblastoma xenograft tumors **A**.-**D**., 1×10^6^ LN229 glioblastoma cells were implanted subcutaneously. After tumor formation, groups were formed. Treatment was started on day 4 and suspended on day 20. Animals were treated intraperitoneally with vehicle (*n* = 9), OTX015 (37.5 mg/kg) (*n* = 9), ABT263 (37.5 mg/kg) (*n* = 9) or both agents *n* = 12) (5 days on, 2 days off). Tumor growth curves show the development of tumor size for each treatment group. Data are presented as mean and SEM. B, Photograph of representative tumors is shown. C, Scatter plots display the quantitative representation of the tumor size among the different treatments toward the end of the experiment. Data are presented as mean and SEM D, Body weights of the animals are provided throughout of the treatment (B). Days are meant as “Days after tumor implantation”. Shown are means. Control group is the same as in A (Vehicle = Control).

In order to assess as to whether or not the synthetic lethal interaction between c-myc and Bcl-xL inhibition can be utilized therapeutically, we employed a heterotopic model of glioblastoma, using p53 mutated LN229 cells. Concerning c-myc inhibition, we chose OTX015 since this compound has entered clinical testing for various malignancies. After tumors were established, four treatment groups were formed that received either vehicle, ABT263 (37.5mg/kg), OTX015 (37.5 mg/kg) or the combination of both 5 days a week. Animals that were treated with the combination treatment displayed significantly smaller tumors than animals receiving vehicle or single treatments (Figure 6A-6C). Moreover, the combination treatment resulted in a regression of tumors ((starting size (mean): 162.6 mm^3^; size at the end of the experiment (mean): 74.6 mm^3^; tumor regression: 54%). Despite the significant anti-glioma *in vivo* efficacy, the combination treatment did not result in any toxicity, which is supported by essentially no weight loss in the combination treatment group throughout application of the drugs (Figure 6D). We also did not appreciate any clinical signs, such as petechiae, that would suggest a medication-induced impairment of coagulation. All in all, these results suggest that our proposed treatment is efficacious and safe.

## DISCUSSION

In this report we demonstrated that simultaneous inhibition of c-myc and Bcl-xL causes synthetic lethality in model systems of one of the most difficult to treat malignancies, which is glioblastoma. We validated this phenomenon both by siRNAs and by pharmacological inhibition. Despite recent promising molecular findings glioblastoma remains an incurable disease with a rapid progression due to resistance to conventional therapies. Therefore, our findings might provide a novel avenue of treatment for this recalcitrant malignancy.

In order to target Bcl-xL and c-myc, we utilized three drug compounds, two of which are in clinical development. The first drug compound is the Bcl-xL inhibitor, ABT263, which is an oral derivative of its predecessor ABT-737 [8] and has reached clinical trials. Early after its discovery, ABT-737 has been tested in preclinical model systems of glioblastoma, which reflected the fact that this compound is working most efficient when administered in combination with other molecules [9]. Another member of this class of molecules is Venetoclax [10] that received accelerated FDA approval recently [11, 12], albeit not for brain tumors. The major advantage of this compound over the others is its improved tolerability in terms of side effects, such as on coagulation [13]. The lack of effect on coagulation can be attributed to the fact that Venetoclax binds preferentially Bcl-2 and to a much lesser degree Bcl-xL [14].

While ABT263 efficiently inhibits Bcl-xL, Bcl-2 and Bcl-w, it fails to bind Mcl-1 [11, 15], which is also commonly up regulated in cancer cells [16]. Consequently, Mcl-1 drives resistance towards BH3-mimetics and therefore ways to counteract Mcl-1 are necessary to take ultimate advantage of BH3-mimetic based therapies. Therefore, research groups are trying to target Mcl-1 by multiple means, e.g. pharmacological compounds, interference with Mcl-1 protein stability or inhibition of Mcl-1 protein synthesis [17–23]. We found here that pharmacological c-myc inhibition by JQ1 [24] decreases Mcl-1 protein levels in multiple glioblastoma cell cultures, including patient derived xenograft cells. Consequently, it is anticipated that JQ1 and OTX015 might have an impact on the sensitivity towards BH3-mimetics. Indeed, careful pharmacological cell death synergism analysis displayed low CI values, indicating a high degree of synergy between ABT263 and JQ1. The combination treatment displays remarkable activity against stem cell-like glioma cells. This might be explained by the earlier findings that stem cell-like glioma cells depend on c-myc signaling for their growth, which is in keeping with our findings that NCH644 and NCH412k stem cell-like glioma cells displayed the highest sensitivity towards JQ1 amongst all cell cultures tested. Therefore, c-myc targeting is likely to hit the stem-cell niche in these neoplasms. To the best of our knowledge, this is the first time that a synthetic lethal interaction between ABT263 and thienotriazolodiazepines has been shown in the setting of malignant glioma. While thienotriazolodiazepines were tested in GBM before, they have not been extensively evaluated in the setting of drug combinations [5]. However, recently, OTX015 was tested in glioblastoma model systems *in vitro* and *in vivo*, demonstrated efficacy and was able to penetrate the blood brain barrier [25]. Moreover, OTX015 acted synergistically with mTOR inhibition and Temozolomide. In this context, the combination treatment of Temozolomide and OTX015 extended animal survival significantly longer than each reagent by its own.

**Figure 7:**
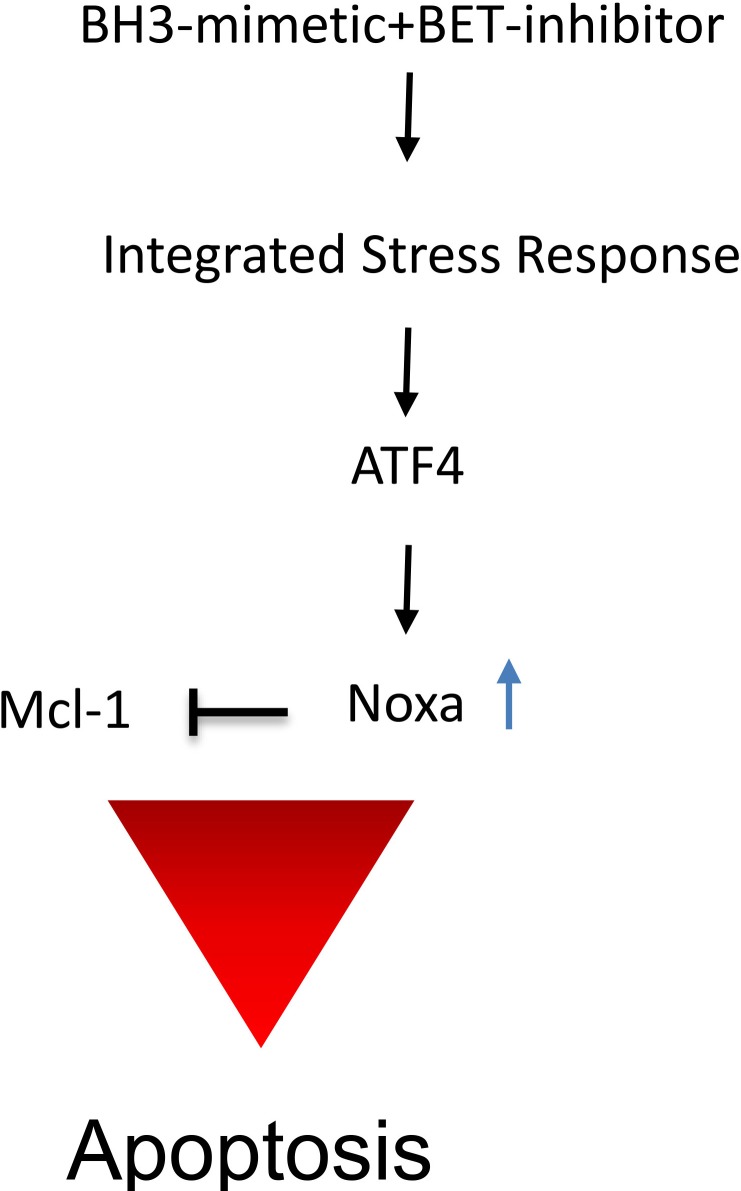
Summary of the proposed mechanism of action by the drug combination The combination treatment of BET-inhibitors and BH3-mimetics causes a stress response with an increase of ATF4 and subsequent up regulation of pro-apoptotic Noxa, interfering with Mcl-1 function, leading to induction of apoptosis.

Our findings revealed that JQ1 elicited a dramatic effect on Bim protein levels. Based on the literature, BIM can be regulated by several pathways, including the JNK/c-Jun/AP-1 pathway as illustrated in [26]. Bim is known to be capable of activating Bax directly [27], which in turn induces intrinsic apoptosis. In all model systems tested, we found a dramatic increase in Bim protein levels [28]. From the present literature, it has been shown that Bim regulates the sensitivity to BH3-mimetics [29]. In that context, high levels of Bim and low levels of Mcl-1 were shown to correlate with sensitivity to ABT263. In our study, Noxa, another pro-apoptotic Bcl-2 family member, was regulated by JQ1 treatment. Especially, the combination treatment of ABT263 and JQ1 led to a significant increase in Noxa levels and knockdown of Noxa [30] by siRNA rescued from the combination treatment of ABT263 and JQ1. Similarly, knockdown of Bak was protective from the combination treatment, suggesting that the therapeutic approach depends not only on Mcl-1 itself, but also on its interacting partners. The increase in Noxa was at the level of transcription. One candidate that regulates Noxa levels is ATF4 [31] and our results confirm the implication of ATF4 in ABT263/JQ1 mediated up regulation of Noxa. The impact of ATF4 on cell death remains controversial, since some reports have suggested a pro-apoptotic, while others favored an anti-apoptotic role [32–35]. Thus far, it appears that the function of ATF4 on cell death appears to be context dependent. Among its other roles, ATF4 is part of the so called integrated stress response and its mRNA translation is increased upon enhanced phosphorylation of eif2α, which can be mediated by the endoplasmic reticulum residing kinase PERK. An important downstream effector of ATF4 is CHOP [36] and in turn CHOP is known to regulate Bim protein levels. A summary of the proposed mechanism of action by the analyzed drug combination is shown in Figure 7.

Finally, we confirmed as to whether or not combined c-myc and Bcl-xL inhibition is efficient in an *in vivo* model system of glioblastoma. For this purpose, we utilized two compounds that are in clinical testing, ABT263 and OTX015. We preferred OTX015 over JQ1, since the latter has poor pharmacokinetics and thus was not considered to be a prime time candidate for clinical testing. Our results indicate that the combination treatment of OTX015 along with ABT263 leads to regression of glioma xenograft tumors without induction of toxicity as demonstrated by no significant weight loss in the treatment group. These results warrant further testing and suggest a reasonable safety profile for this treatment approach in patients.

## MATERIALS AND METHODS

### Reagents

ABT263, JQ1 and OTX015 were purchased from Selleckchem (Houston, TX). A 10 mM working solution in dimethylsulfoxide (DMSO) was prepared for all reagents prior to storage at -20°C. Final concentrations of DMSO were below 0.1% (v/v).

### Cell cultures and growth conditions

All cells were cultured as described [17, 37–41]. GBM6, GBM14 and GBM39 human, patient-derived glioblastoma xenograft cultures originated from Dr. Jann Sarkaria (Mayo Clinic, Rochester, MI, U.S.A.) and were cultured in serum-containing media with DMEM. The identities of the glioblastoma cell lines we purchased were confirmed by the respective source of purchase.

NCH644 and NCH421K glioma stem-like cells were cultured in MG-43 medium (CLS, Heidelberg, Germany) for both maintenance and experiments [17, 37–41].

### Cell viability assays

In order to examine cellular proliferation, CellTiter-Glo^®^ assays were performed as previously described [17, 18, 42].

### Measurement of apoptosis and mitochondrial membrane potential

Annexin V/propidium iodide, propidium iodide and TMRE staining were performed as previously described or in accordance with the manufacturer instructions for TMRE staining (Cell Signaling) [43]. The data were analysed with the FlowJo software (version 8.7.1; Tree Star, Ashland, OR).

### Western blot analysis

Specific protein expression in cell lines was determined by Western blot analysis as described before [44]. The following antibodies were used: Mcl-1 (1:500; CST: Cell Signaling Technology, Danvers, MA), human caspase-9 (1:1,000; CST), cleaved caspase 3 (1:250; CST), cleaved PARP (Asp214, 1:1000; CST), Bak (1:500; CST), Bcl-2 (1:500; CST), BIM (1:500; CST), ATF4 (1:500; CST), c-myc (1:500; CST), Bcl-xL (1:500; CST), Usp9X (1:1000; CST), Noxa (1:500, clone 114C307; Calbiochem), β-actin (1:2,000, clone AC15; Sigma Aldrich) and secondary HRP-linked antibodies were purchased from Santa Cruz Biotechnology Inc. Some western blots were acquired, using the Azure (C300) imaging system (CCD – camera based).

### Transfections of siRNAs

Transfections were performed as previously described [43], using either Oligofectamine or Lipofectamine 2000. PMAIP1 siRNA and BAK siRNA were purchased from Ambion. The two Bcl-xL and two c-myc specific siRNAs were purchased from CST: Cell Signaling Technology, Danvers, MA. Non-targeting siRNA-pool (ON-TARGETplus Non-targeting Pool, # D-001810-10-05), ATF4 (SMARTpool: ON-TARGETplus ATF4 siRNA, L-005125-00-0005) and four individual Mcl-1 siRNAs (ON-TARGETplus Mcl-1 siRNAs; siRNA-1: GGU UUG GCA UAU CUA AUA A; siRNA-2: GAA GGU GGC AUC AGG AAU G, siRNA-3: GAU UAU CUC UCU CGG UAC CUU, siRNA-4: CGA AGG AAG UAU CGA AUU U (Dharmacon)) were purchased from Thermo Fisher Scientific.

### Real-time PCR and cDNA synthesis

RT-PCR and cDNA synthesis was performed as described before [44], using the following primers. 18S forward: AGT CCC TGC CCT TTG TAC ACA, 18S reverse: GAT CCG AGG GCC TCA CTA AAC, PMAIP1 forward: CTG GAA GTC GAG TGT GCT ACT C, PMAIP1 (Noxa) reverse: TGA AGG AGT CCC CTC ATG CAA G. The analysis of the results was performed as previously described [44].

### Subcutaneous xenograft model

1 × 10^6^ LN229 glioblastoma cells (p53 mutated) suspended 1:1 in Matrigel® Matrix (Corning Inc., Corning, NY) were implanted subcutaneously into the flanks of 6-8 week-old Nu/Nu mice as described before [18–20]. Tumors were measured with a caliper and sizes calculated according to the standard formula: (length * width^2^)*0.5. Treatment was performed intraperitoneally. For intraperitoneal application ABT263 and OTX015 were dissolved in 10% DMSO, 32% Cremophor EL (SIGMA, St. Louis, MO), 8% Ethanol (Pharmco-Aaper, Brookfield,CT) and 50% PBS.

### Statistical analysis

Statistical significance was assessed by two-tailed Student's t-test using Prism version 5.04 (GraphPad, La Jolla, CA). A p ≤ 0.05 was considered statistically significant. The CompuSyn software (ComboSyn, Inc., Paramus, NJ - www.combosyn.com last accessed 06/01/15) was used for the drug combination analysis including the calculation of the combination index (CI) [40]. A CI < 1 was considered as synergistic, a CI = 1 as additive and a CI > 1 as antagonistic.

### Study approval

All procedures were in accordance with Animal Welfare Regulations and approved by the Institutional Animal Care and Use Committee at the Columbia University Medical Center.

## SUPPLEMENTARY MATERIALS FIGURES AND TABLES


