# Supplementary material for: BH3-mimetics and BET-inhibitors elicit enhanced lethality in malignant glioma
Source: Oncotarget. 2017 Mar 18;8(18):29558–73. doi: 10.18632/oncotarget.16365 (PMC5444687; doi:10.18632/oncotarget.16365)
Supplement: Supplementary file 1 [file oncotarget-08-29558-s001.pdf]

BH3-mimetics and BET-inhibitors elicit enhanced lethality in malignant glioma

Supplementary Material

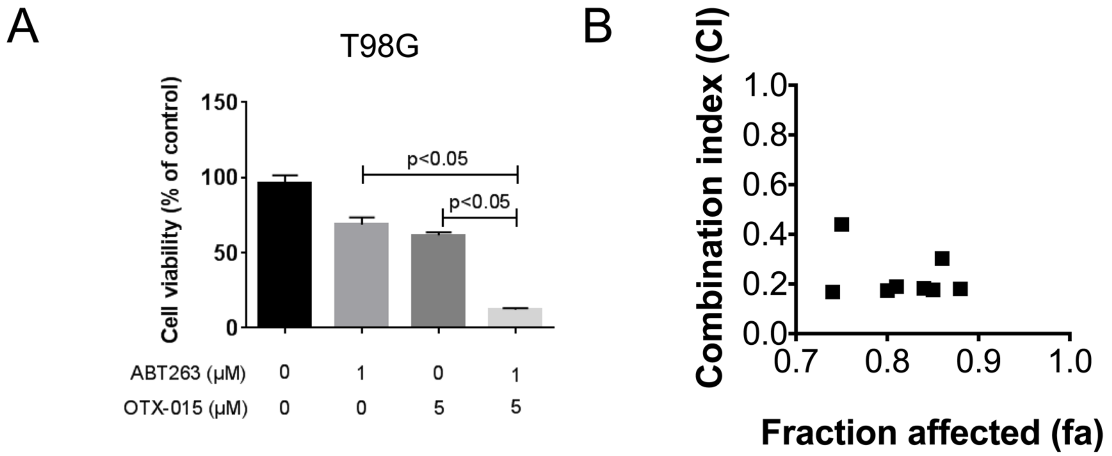

Supplementary Figure 1: The combination treatment of ABT263 and OTX015 causes synergistic reduction in cellular viability. A, T98G cells were treated with solvent, ABT263, OTX015 or the combination of both for 72h. Subsequently, T98G cells were analyzed by Cell-Titer Glo assay. P-values were calculated and a p value of less than 0.05 was considered statistically significant. Columns: mean, Error bar: standard deviation. B, NCH421k glioblastoma cells were treated with ABT263, JQ1 or the combination for 72 hours. Subsequently, NCH421k cells were analyzed by Cell-Titer Glo assay. CI values and fraction affected were calculated using the CompuSyn software (ComboSyn, Inc., Paramus, NJ, U.S.A.). Data points located below 1 (CI value less than 1) indicate a synergistic drug-drug interaction and data points larger than 1 indicate an antagonistic drug-drug interaction.

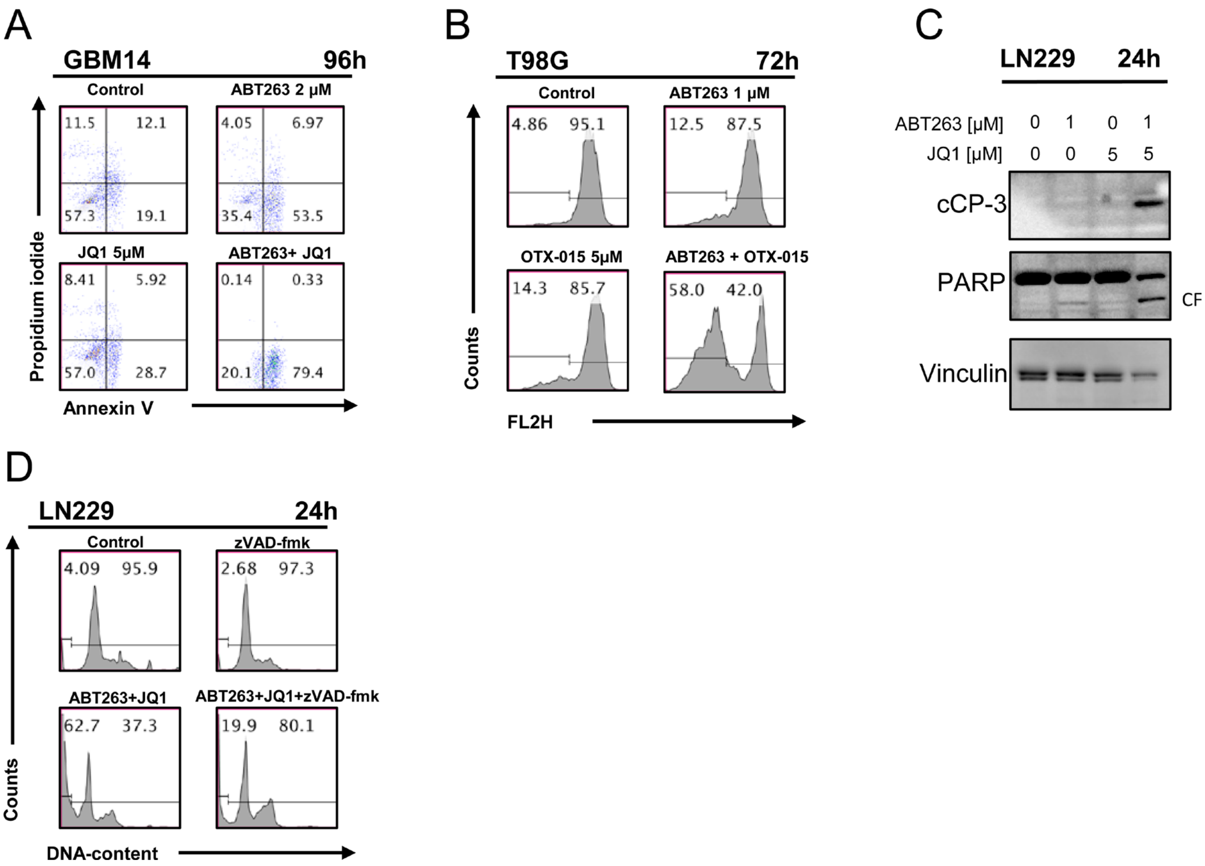

Supplementary Figure 2: The combination treatment of ABT263+JQ1 or OTX015 elicits enhanced activation of apoptosis and dissipation of mitochondrial membrane potential. A, GBM14 cells were treated with solvent, ABT263, JQ1 or the combination for 96 h. Cells were stained with Annexin V/Propidium iodide and analyzed by flow cytometry. B, T98G cells were treated with solvent, ABT263, JQ1 or the combination for 72h. Cells were stained with TMRE and analyzed by flow cytometry. C, LN229 cells were treated with solvent, ABT263, JQ1 or the combination for 24h. Cell lysates were prepared and analyzed by western blotting for cleaved caspase-3 (cCP-3), PARP and Vinculin (loading). CF: cleaved fragment of PARP (89 kDa). D, LN229 cells were treated with the combination of ABT263 (2 µM) and JQ1 (5 µM) in the presence or absence of zVAD-fmk (20 µM) for 24 hours. Subsequently, cells were harvested, fixed and stained with Propidium iodide and analyzed for DNA fragmentation (flow cytometry).

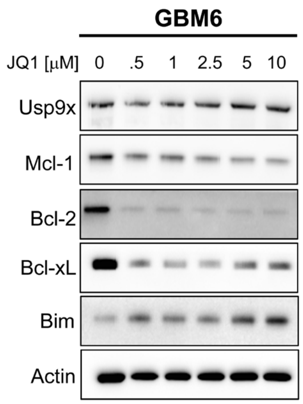

Supplementary Figure 3: GBM6 cells were treated with increasing concentrations of JQ1 for 72h. Cell lysates were prepared and analyzed by western blotting for the expression of Usp9x, Mcl-1, Bcl-2, Bcl-xL, Bim and Actin.

Supplementary Table 1: ABT263 treatment elicits a synergistic anti-proliferative effect on NCH421k glioma cells and NCH644 stem cell-like glioma cells in the presence of the c-myc inhibitor, JQ1.

NCH421K		
**ABT263 (μM)**	**JQ1 (μM)**	**CI**
0.25	4.0	0.18051
0.5	2.0	0.18271
1.0	1.0	0.18913
2.0	0.5	0.44002
2.0	4.0	0.30280
1.0	2.0	0.17678
0.5	1.0	0.17324
0.25	0.5	0.16823

NCH421k cells were treated and analyzed as described in supplementary Figure 1B. The CompuSyn software (ComboSyn, Inc., Paramus, NJ) was used for the drug-drug interaction analysis including the calculation of the combination index (CI). A CI<1 was considered as synergistic, a CI=1 as additive and a CI>1 as antagonistic.
